# The Outcome of Prophylactic Intravenous Cefazolin and Ceftriaxone in Cirrhotic Patients at Different Clinical Stages of Disease after Endoscopic Interventions for Acute Variceal Hemorrhage

**DOI:** 10.1371/journal.pone.0061666

**Published:** 2013-04-22

**Authors:** Cheng-Kun Wu, Jing-Houng Wang, Chen-Hsiang Lee, Keng-Liang Wu, Wei-Chen Tai, Sheng-Nan Lu, Tsung-Hui Hu, Seng-Kee Chuah

**Affiliations:** 1 Division of Hepato-gastroenterology, Department of Internal Medicine, Kaohsiung Chang Gung Memorial Hospital and Chang Gung University, College of Medicine, Kaohsiung, Taiwan; 2 Division of Infectious Disease, Department of Internal Medicine, Kaohsiung Chang Gung Memorial Hospital and Chang Gung University, College of Medicine, Kaohsiung, Taiwan; University of Colorado, United States of America

## Abstract

Antibiotic prophylaxis with norfloxacin, intravenous ciprofloxacin, or ceftriaxone has been recommended for cirrhotic patients with gastrointestinal hemorrhage but little is known about intravenous cefazolin. This study aimed to compare the outcome of intravenous cefazolin and ceftriaxone as prophylactic antibiotics among cirrhotic patients at different clinical stages, and to identify the associated risk factors. The medical records of 713 patients with acute variceal bleeding who had received endoscopic procedures from were reviewed. Three hundred and eleven patients were entered for age-matched adjustment after strict exclusion criteria. After the adjustment, a total of 102 patients were enrolled and sorted into 2 groups according to the severity of cirrhosis: group A (Child’s A patients, n = 51) and group B (Child’s B and C patients, n = 51). The outcomes were prevention of infection, time of rebleeding, and death. Our subgroup analysis results failed to show a significant difference in infection prevention between patients who received prophylactic cefazolin and those who received ceftriaxone among Child’s A patients (93.1% vs. 90.9%, p = 0.641); however, a trend of significance in favor of ceftriaxone prophylaxis (77.8% vs. 87.5%, p = 0.072) was seen among Child’s B and C patients. More rebleeding cases were observed in patients who received cefazolin than in those who received ceftriaxone among Child’s B and C patients (66.7% vs. 25.0%, p = 0.011) but not in Child’s A patients (32% vs. 40.9%, p = 0.376). The risk factors associated with rebleeding were history of bleeding and use of prophylactic cefazolin among Child’s B and C patients. In conclusion, this study suggests that prophylactic intravenous cefazolin may not be inferior to ceftriaxone in preventing infections and reducing rebleeding among Child’s A cirrhotic patients after endoscopic interventions for acute variceal bleeding. Prophylactic intravenous ceftriaxone yields better outcome among Child’s B and C patients.

## Introduction

Multiple clinical trials have shown an overall reduction in infectious complications and decreased mortality in cirrhotic patients with gastrointestinal bleeding who are receiving prophylactic antibiotics [1]–[6]. Antibiotics also reduce the incidence of rebleeding in cirrhotic patients who had bled from esophageal varices [7]. Previous studies have shown that enteric aerobic gram-negative bacteria are the most common causative organisms of gastrointestinal bleeding in cirrhotic patients [1], [3], [8]. Both the American Association for the Study of Liver Disease (AASLD) and the Baveno V consensus recommended antibiotic prophylaxis for cirrhotic patients with upper gastrointestinal bleeding [9], [10]. Oral norfloxacin (400 mg twice daily), intravenous ciprofloxacin, and intravenous ceftriaxone (1 g/day) are preferred. However, in case of a high prevalence of quinolone-resistant organisms, intravenous ceftriaxone is more effective than fluoroquinolone [11].

The first-generation cephalosporins are predominantly used against a wide range of bacterial species, including community-acquired strains of *Escherichia coli* and *Klebsiella pneumoniae* [12]. Our previous study showed that the use of intravenous cefazolin in cirrhotic patients with acute variceal hemorrhage after endoscopic interventions could effectively reduce infections, and revealed a trend of actuarial probability of remaining free of early rebleeding [13]. Theoretically, cefazolin may have a similar effect as ceftriaxone in cirrhotic patients with gastrointestinal bleeding. This study aimed to compare the outcome of intravenous cefazolin and ceftriaxone as prophylactic antibiotics in a prospective registered cohort of cirrhotic patients at different clinical stages of disease who had acute variceal hemorrhage after endoscopic interventions, and to identify the associated confounding factors relevant to the outcome.

## Materials and Methods

### Patients

From July 2009 to April 2012, the medical records of 713 patients with acute variceal bleeding who had received endoscopic procedures from a university-affiliated tertiary care center were reviewed. We excluded patients with unsuccessful endoscopic hemostasis, incomplete chart records, or insufficient follow-up period (<30 days); patients who already had signs of infections (body temperature >38°C, white blood cells >10,000/µL); patients with occult infection (defined as positive blood cultures obtained before antibiotic prophylaxis); and patients using other kinds of antibiotics before endoscopy. Eventually, a total of 311 patients were entered for age-matched adjustment. After the adjustment, a total of 102 patients (male/female, 66∶36; age, 60.4±13.2 years) were enrolled and sorted into 2 groups according to the severity of liver cirrhosis: group A (Child’s A patients, n = 51) and group B (Child’s B and C patients, n = 51). Intravenous cefazolin (1 g, q8 h) for 2–7 days or intravenous ceftriaxone (1 g, q12 h) for 2–7 days was prescribed as the prophylactic antibiotic. The choice of antibiotic, dose, and duration of therapy were determined by the clinicians. The end points were the incidence of infections, time of rebleeding, and death (during hospitalization). In addition, we performed a subgroup analysis for both Child’s A cirrhotic patients and patients with advanced cirrhosis (Child’s B and C). This retrospective chart review study was approved by both the institutional review board and the ethics committee of Chang Gung Memorial Hospital, Taiwan (101–2170B). All patients provided written informed consent before the endoscopic interventions. None of our patients were minors or children.

### Definitions

Cirrhosis was diagnosed according to clinical, laboratory, abdominal ultrasonographic [14], and/or histological findings. Its severity was classified according to Pugh’s modification of Child’s classification [15]. Esophageal variceal or gastric variceal bleeding was diagnosed according to the following: 1) clinical signs of hematemesis, coffee-ground vomitus, hematochezia, or melena; 2) endoscopic signs of active bleeding, adherent blood clots, white nipple signs, or erosions on varices; and/or 3) large varices with a red color sign without other bleeding sources. Vital signs were checked and laboratory tests, including white blood cell count, hemoglobin, platelet count, prothrombin time, albumin, and bilirubin levels, were obtained when cirrhotic patients with acute gastrointestinal hemorrhage arrived at the emergency room (ER). Two sets of blood culture were obtained before administrating antibiotics. Terlipressin (Glypressin) or octreotide (Sandostatin) was administered for 3 days. A nasogastric tube, 2 intravenous catheters, or central venous catheters were placed as clinically indicated. Patients underwent endoscopic procedures within 24 hours of arrival at the ER. Endoscopic variceal ligation or endoscopic variceal injection sclerotherapy was performed on the patients by experienced endoscopists.

The diagnosis of spontaneous bacterial peritonitis was based on ≥250 neutrophils/µL in ascetic fluid. The diagnosis of urinary tract infection, pneumonia, and bacteremia was made according to the definitions from the Centers for Disease Control and Prevention, 2013 [16]. Rebleeding was defined as a new onset of hematemesis, coffee-ground vomitus, hematochezia, or melena, with an increasing pulse rate of >110 beats per minute and decreasing blood pressure of <90 mm Hg after a 24-hour period of stable vital signs and hematocrit levels following endoscopic treatment. Early rebleeding was defined as recurrent bleeding that occurred in <7 days.

### Statistical Analysis

All results are expressed as means ± standard deviations for continuous data and as frequencies or percentages for categorical data. Distributions of continuous variables were analyzed by the independent-sample t test. Kaplan-Meier analysis with log-rank test was used to compare the differences in infection and rebleeding among groups. Variables were analyzed using the multivariate Cox proportional hazard model to determine the independent predictive factors of infection and rebleeding. Only the variables significant in univariate analysis were entered in the multivariate analysis. The results were expressed as hazard ratios (HR) with 95% confidence intervals (95% CIs). Statistical significance was taken as p<0.05. All analyses were performed using SPSS ver. 18 (SPSS Inc., Chicago, IL, USA).

## Results

### Demographic and Clinical Characteristics

The clinical and laboratory characteristics of the patients are summarized in Table 1. A total of 102 patients (66 men and 36 women; mean age, 60.4±13.2 years) were enrolled. Among them, cirrhosis was diagnosed by a liver biopsy in 18 patients; in other patients, cirrhosis was diagnosed by abdominal ultrasonography and clinical and laboratory data. Thirty-six patients (35.2%) had hepatitis B virus (HBV) infection, 35 (34.3%) had HCV, 27 (26.4%) had alcohol-related cirrhosis, 2 (1.9%) had alcohol- and HBV-related cirrhosis, 1 (0.98%) had HBV and HCV dual infection, and 1 (0.98%) had alcohol- and HCV-related cirrhosis. The 2 groups had comparable clinical and laboratory data, except for the higher total bilirubin level, lower albumin level, and more prolonged prothrombin time in group B patients.

**Table 1:** Clinical characteristics of the 2 groups of patients.

Characteristics	Group A (Child’s A patients)(n = 51)	Group B (Child’s B+C patients) (n = 51)	*p* Value
Age (years)	60.5±13.9	60.3±12.6	0.959
Male sex, n (%)	32 (62.7)	34 (66.7)	0.679
Etiology			
Alcohol-related, n (%)	14 (27.5)	16 (31.4)	0.826
HBV, n (%)	21 (41.2)	18 (35.3)	0.541
HCV, n (%)	19 (34.5)	18 (35.3)	0.837
Vitals at the ER			
BT (°C)	36.5±0.4	36.4±0.4	0.260
HR (beats/min)	87.8±15.7	89.8±14.1	0.489
SBP (mm Hg)	128.8±28.9	127.9±30.9	0.882
Laboratory			
WBC (×10^9^/L)	5.8±2.0	6.5±2.5	0.121
Hb (g/dL)	9.7±2.1	9.2±1.6	0.171
PLT (10^9^/L)	88.3±45.0	80.2±33.1	0.298
PT (S)	12.5±1.1	13.4±1.7	0.003
Albumin (g/L)	3.2±0.4	2.8±0.5	<0.001
Bilirubin (mg/dL)	1.4±0.7	2.6±2.6	0.002
Prior bleeding event, n (%)	9 (17.6)	15 (29.4)	0.161
Medication before bleeding β-Blocker, n (%)	18 (35.3)	25 (49.0)	0.160
Acute bleeding Glypressin, n (%)	50 (98)	51 (100)	0.315
Octreotide, n (%)	1 (2)	0 (0)	0.315
Cefazolin/Ceftriaxone, n (%)	29 (56.9)/22 (43.1)	27 (52.9)/24 (47.1)	0.691
EV/GV, n (%)	42 (82.4)/9 (17.6)	43 (84.3)/8 (15.7)	0.790
Hospital days	11.0±10.3	11.4±9.1	0.143

**Abbreviations:** HBV, hepatitis B virus; HCV, hepatitis C virus; ER, emergency room; BT, body temperature.

HR, heart rate; SBP, systolic blood pressure; WBC, white blood cells; Hb, hemoglobin; PLT, platelet count;

PT, prothrombin time; EV, esophageal varices; GV, gastric varices.

### Bacterial Infections

**Figure 1:**
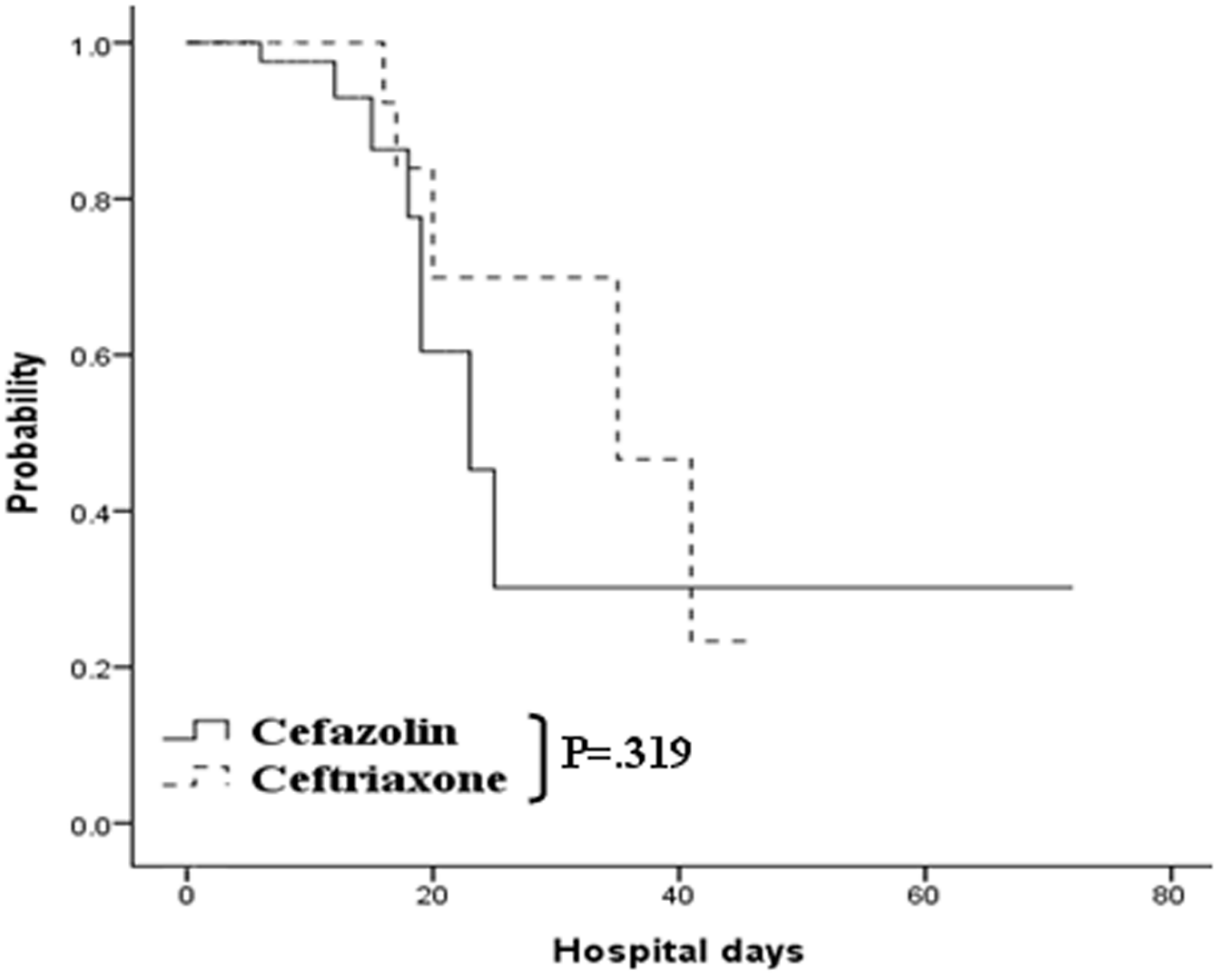
Actuarial probability of remaining free of infection in cirrhotic patients at all stages. No statistically significant difference was observed between the cefazolin and ceftriaxone groups (*p* = 0.319).

**Figure 2:**
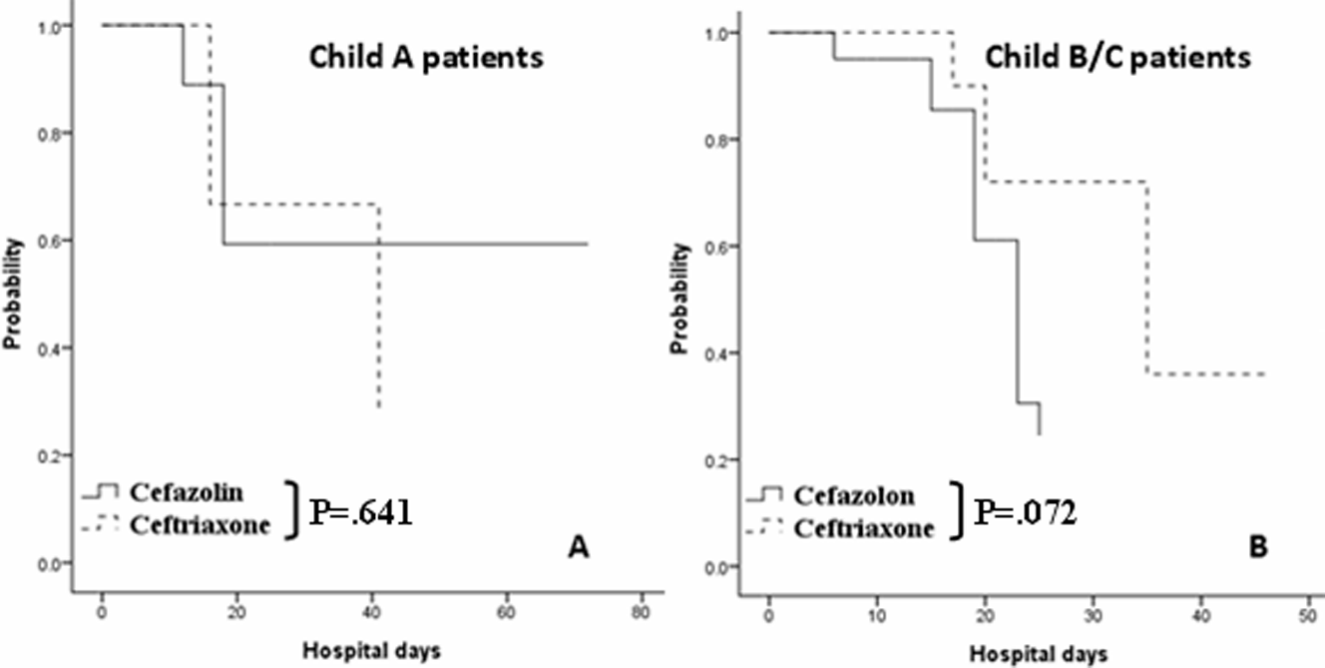
Actuarial probability of remaining free of infection at different clinical stages of cirrhotic patients. There was a similar probability of remaining free of infection between patients who were prescribed with intravenous cefazolin and those prescribed with ceftriaxone in Child’s A group (p = 0.641 by log-rank test) (A). A trend of significance was observed in favor of patients prescribed with prophylactic ceftriaxone in Child’s B and C group (*p = *0.072) (B).

The outcomes of infections are summarized in Table 2. Of the proved infections, pneumonia was the predominant etiology. *Escherichia coli, K. pneumoniae*, and *Pseudomonas aeruginosa* were the organisms isolated from patients who had positive bacterial cultures. The outcome analysis failed to show a significant difference in infection prevention between patients who received prophylactic intravenous cefazolin and those who received intravenous ceftriaxone among all cirrhotic patients (85.7% vs. 89.1%, p = 0.319) (Figure 1). The same result was obtained in the subgroup analysis for Child’s A patients (93.1% vs. 90.9%, *p* = 0.641) (Figure 2A); however, a trend of significance was observed in favor of those who received prophylactic ceftriaxone in Child’s B and C patients (77.8% vs. 87.5%, *p* = 0.072) (Figure 2B). Univariate analysis showed that age, Child’s B and C disease status, thrombocytopenia, hypoalbuminemia, hyperbilirubinemia, and prothrombin time prolongation were the significant confounding factors relevant to infection. Multivariate analysis identified 3 independent predictors for infection: age (HR, 1.060; 95% CI, 1.008–1.115; *p = *0.022), hypoalbuminemia (HR, 0.135; 95% CI, 0.032–0.570; p = 0.006), and hyperbilirubinemia (HR, 1.382; 95% CI, 1.001–1.908; p = 0.049) (Table 3).

**Table 2:** Outcomes of infections in the 2 groups of patients.

Characteristics	Group A (Child’s A patients) (n = 51)	Group B (Child’s B+C patients) (n = 51)	*p* Value
Infections, n (%)	4 (7.8)	9 (17.6)	0.139
Bacteremia	0	1 (2.0)	0.315
Pneumonia	4 (7.8)	7 (13.7)	0.338
UTI	0	0	
SBP	0	1 (2.0)	0.315
Organisms, n (%)	1 (2.0)	3 (5.9)	0.308
*E. coli*	1 (2.0)	1 (2.0)	1.000
*K.P*	0	1 (2.0)	0.315
*PS*	0	1 (2.0)	0.315

**Abbreviations:** UTI, urinary tract infection; SBP, spontaneous bacterial peritonitis*; E. coli, Escherichia coli*; *K.P, Klebsiella pneumonia*; *PS*, *Pseudomonas aeruginosa.*

**Table 3:** Univariate and multivariate analyses of potential risk factors for infection in patients with cirrhosis and variceal bleeding following endoscopic treatment.

Variable	Infected cases	Univariate analysis		Multivariate analysis	
	N = 13	Hazard ratio (95% CI)	*p* Value	Hazard ratio (95% CI)	*p* Value
Age		1.055 (1.011–1.101)	0.014	1.060 (1.008–1.115)	0.022*
Male sex	7 (53.8%)	1.810 (0.630–5.193)	0.270		
Etiology of liver cirrhosisAlcohol-related	4 (30.8%)	0.698 (0.208–2.346)	0.561		
HBV	6 (46.2%)	1.159 (0.401–3.349)	0.785		
HCV	4 (30.8%)	1.283 (0.443–3.717)	0.646		
Child-Pugh class B/C	9 (69.2%)	2.831 (0.917–8.738)	**0.070**		0.809
WBC (10^9^/L)		1.127 (0.900–1.410)	0.297		
PLT (10^9^/L)		1.000 (0.987–1.013)	0.984		
PT (s)		1.439 (1.054–1.965)	0.022		0.805
Albumin (g/L)		0.106 (0.026–0.423)	0.001	0.135 (0.032–0.570)	0.006**
Total bilirubin		1.306 (1.012–1.686)	0.040	1.382 (1.001–1.908)	0.049***
Recurrent bleeding	6 (46.2%)	1.008 (0.342–2.972)	0.779		
Antibiotic choice (cefazolin)	8 (61.5%)	1.630 (0.552–4.809)	0.376		

**Abbreviations:** CI, confidence interval; HBV, hepatitis B virus; HCV, hepatitis C virus; WBC, white blood cells; Hb, hemoglobin; PLT, platelet count; PT, prothrombin time. *p* = *0.022, **p* = *0.006, ***p* = *0.049.

### Rebleeding and Mortality

**Figure 3:**
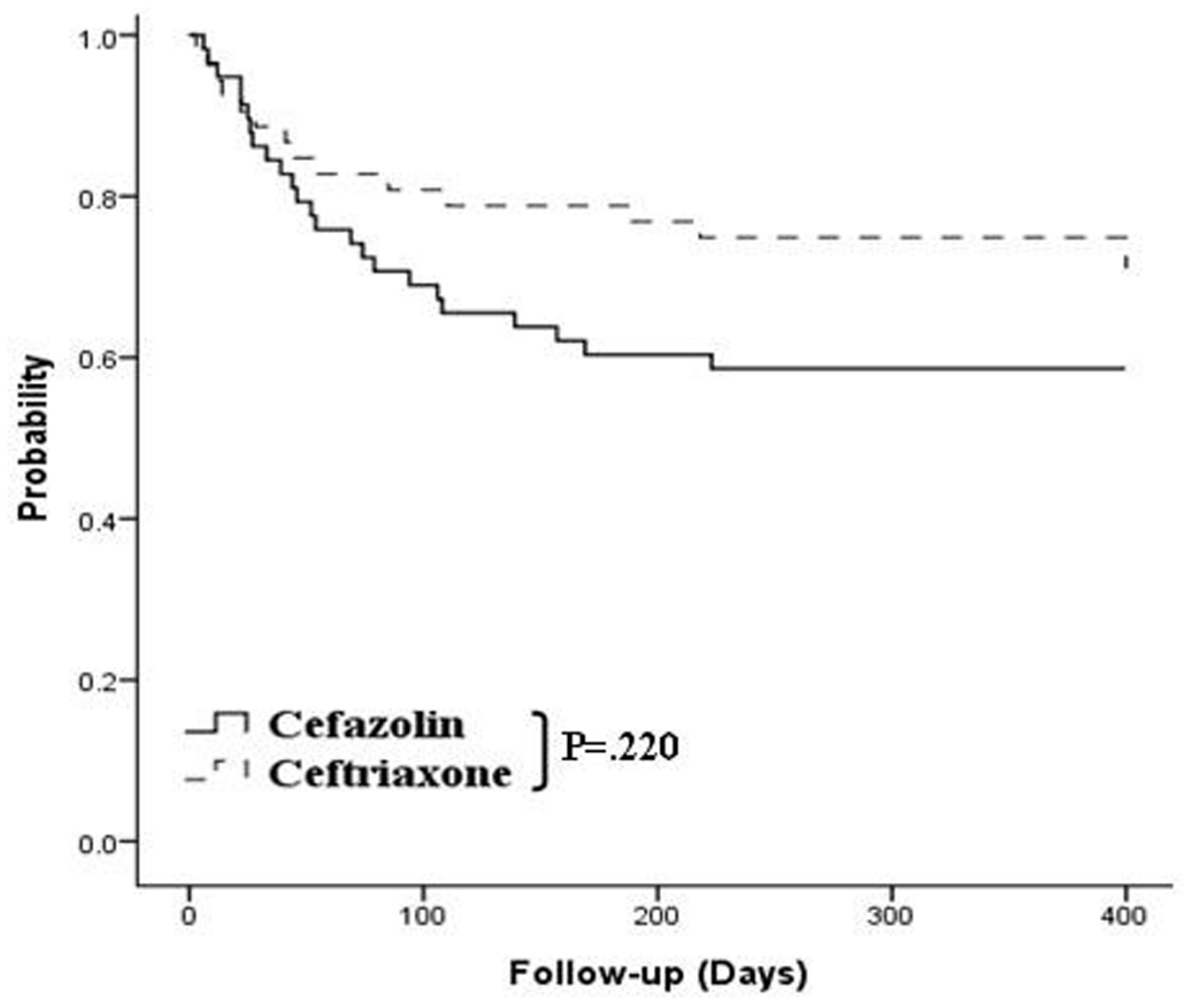
Actuarial probability of remaining free of rebleeding in cirrhotic patients at all stages. No statistically significant difference was observed between the cefazolin and ceftriaxone groups (*p = *0.220).

**Figure 4:**
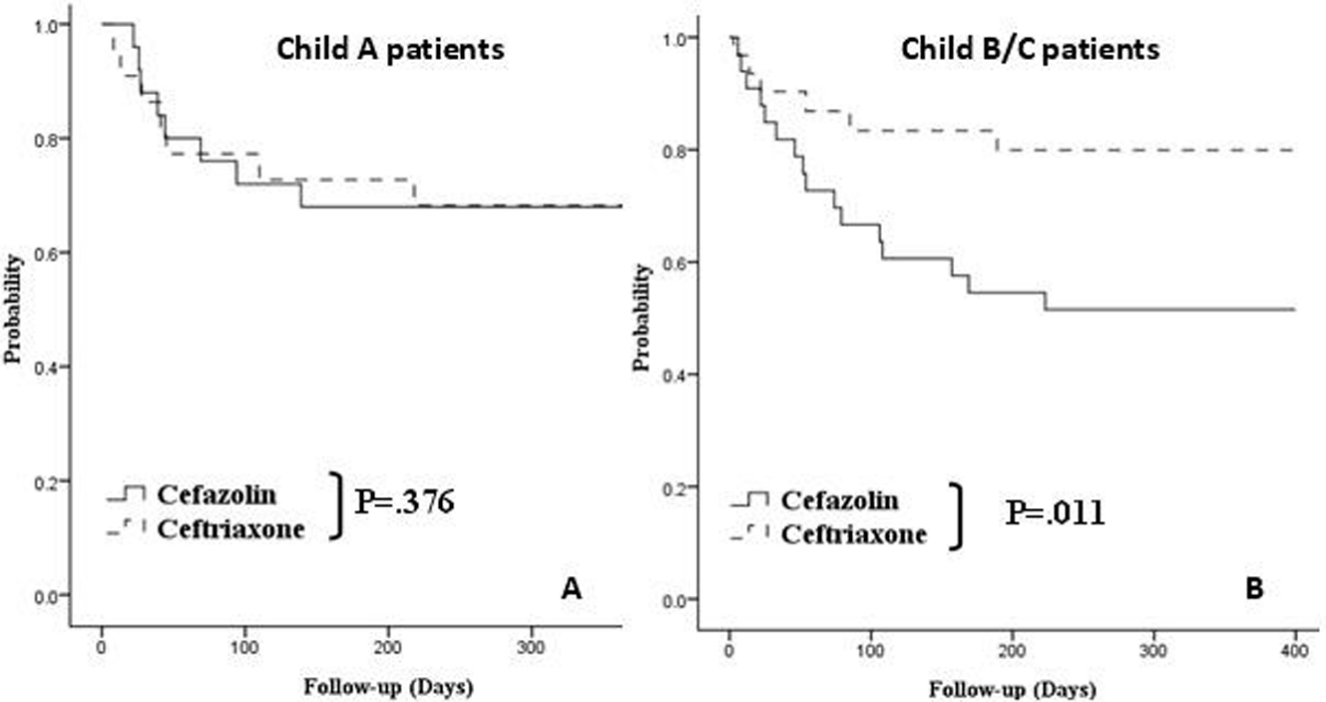
Actuarial probability of remaining free of rebleeding at different clinical stages of cirrhotic patients. There was a similar probability of remaining free of rebleeding between patients who were prescribed with intravenous ceftriaxone and those prescribed with cefazolin in Child’s A group (p = 0.376 by log-rank test) (A). A significantly higher probability of remaining free of rebleeding was observed in those who were prescribed with intravenous cefazolin than in those given ceftriaxone in Child’s B and C group (*p* = 0.011) (B).

The hemostatic outcomes of all the patients are summarized in Table 4. As shown in Figure 3, there was no significant difference in the actuarial probability of remaining free of overall rebleeding between patients prescribed with cefazolin and those prescribed with ceftriaxone, before subgroup analysis according to disease severity (*p* = 0.220). The independent risk factors were thrombocytopenia (HR, 0.992; 95% CI, 0.985–0.999; *p = *0.029) and history of bleeding (HR, 2.674; 95% CI, 1.348–5.305; *p* = 0.005) (Table 5). Although we failed to show a significant difference between patients who received prophylactic intravenous cefazolin and those who received ceftriaxone among Child’s A patients (32% vs. 40.9%, *p = *0.376) (Figure 4A), after performing subgroup analysis according to disease severity, we observed more rebleeding in patients who received prophylactic cefazolin among Child’s B and C patients (66.7% vs. 25.0%, *p = *0.011) (Figure 4B). The risk factors associated with rebleeding were a history of bleeding (HR, 2.069; 95% CI, 0.908–4.714; *p = *0.084) and the use of prophylactic cefazolin instead of ceftriaxone among Child’s B and C patients (HR, 2.896; 95% CI, 1.141–7.349; p = 0.025) (Table 6).

**Table 4:** Hemostatic outcome in patients with variceal bleeding following endoscopic treatment.

	Group A (Child’s A patients) (n = 51)	Group B (Child’s B+C patients) (n = 51)	*p* Value
No. of rebleeding, n (%)	17 (33.3)	24 (47.1)	0.157
Time of rebleeding			
Early (≤6 weeks), n (%)	10 (19.6)	11 (21.6)	0.807
<7 days, n (%)	2 (3.9)	3 (5.9)	0.647
8–14 days, n (%)	2 (3.9)	3 (5.9)	0.647
15–42 days, n (%)	6 (11.8)	5 (9.8)	0.750
Late (>6 weeks), n (%)	7 (13.7)	13 (25.5)	0.135

**Table 5:** Univariate and multivariate analyses of potential risk factors for rebleeding in all patients with cirrhosis and variceal bleeding following endoscopic treatment (before subgroup analysis).

Variable	Univariate analysis		Multivariate analysis	
	Hazard ratio (95% CI)	*p* Value	Hazard ratio (95% CI)	*p* Value
Age	0.993 (0.970–1.017)	0.412		
Male sex	1.169 (0.613–2.231)	0.636		
Etiology of liver cirrhosisAlcohol-related	0.842 (0.422–1.681)	0.626		
HBV	1.261 (0.677–2.348)	0.465		
HCV	0.660 (0.337–1.294)	0.226		
Child-Pugh class B/C	1.513 (0.813–2.818)	0.191		
β-Blocker use	0.813 (0.431–1.536)	0.524		
Hb (g/dL)	0.856 (0.734–0.999)	0.048		
PLT (10^9^/L)	0.993 (0.985–1.000)	0.045	0.992 (0.985–0.999)	0.029*
PT (s)	0.826 (0.664–1.028)	0.086		
Albumin (g/L)	0.846 (0.477–1.501)	0.567		
Total bilirubin	0.990 (0.848–1.155)	0.895		
Prior bleeding	2.081 (1.089–3.980)	**0.027**	2.674 (1.348–5.305)	0.005**
Bacterial infection	1.173 (0.493–2.790)	0.718		
Antibiotic choice (cefazolin)	1.481 (0.784–2.797)	0.226		

**Abbreviations:** CI, confidence interval; HBV, hepatitis B virus; HCV, hepatitis C virus; WBC, white blood cells; Hb, hemoglobin; PLT, platelet count; PT, prothrombin time.

* p = 0.029, **p = 0.005.

**Table 6:** Univariate and multivariate analyses of potential risk factors for rebleeding in patients with Child’s A and Child’s B and C variceal bleeding following endoscopic treatment (after subgroup analysis).

Variable	Univariate analysis		Multivariate analysis	
	Hazard ratio (95% CI)	*p* Value	Hazard ratio (95% CI)	*p* Value
Child’s A patients				
Age	1.016 (0.980–1.052)	0.387		
Male sex	0.823 (0.313–2.163)	0.693		
Etiology of liver cirrhosisAlcohol-related	0.486 (0.140–1.693)	0.257		
HBV	1.407 (0.542–3.655)	0.483		
HCV	0.900 (0.333–2.435)	0.835		
β-Blocker use	1.134 (0.419–3.071)	0.804		
WBC (10^9^/L)	0.848 (0.663–1.086)	0.191		
Hb (g/dL)	0.838 (0.678–1.035)	0.100		
PLT (10^9^/L)	0.999 (0.982–1.001)	0.097		
PT (s)	0.656 (0.415–1.036)	0.071		
Albumin (g/L)	0.581 (0.212–1.595)	0.292		
Total bilirubin	0.409 (0.156–1.075)	0.070		
Prior bleeding	1.559 (0.508–4.788)	0.438		
Bacterial infection	1.284 (0.293–5.623)	0.740		
Antibiotic choice (cefazolin)	0.653 (0.252–1.693)	0.381		
Child’s B/C patients				
Age	0.972 (0.940–1.005)	0.101		
Male sex	1.566 (0.648–3.783)	0.319		
Etiology of liver cirrhosisAlcohol-related	1.212 (0.518–2.834)	0.658		
HBV	1.161 (0.508–2.654)	0.723		
HCV	0.522 (0.207–1.316)	0.168		
β-Blocker use	0.504 (0.260–1.358)	0.217		
WBC (10^9^/L)	1.041 (0.885–1.225)	1.041		
Hb (g/dL)	0.894 (0.703–1.137)	0.361		
PLT (10^9^/L)	0.992 (0.979–1.004)	0.206		
PT (s)	0.847 (0.669–1.072)	0.168		
Albumin (g/L)	1.353 (0.626–2.923)	0.442		
Total bilirubin	0.993 (0.856–1.152)	0.926		
Prior bleeding	2.306 (1.108–5.223)	**0.045**	2.069 (0.908–4.714)	0.084
Bacterial infection	1.039 (0.355–3.040)	0.945		
Antibiotic choice (cefazolin)	3.104 (1.229–7.835)	0.017	2.896 (1.141–7.349)	0.025*

**Abbreviations:** CI, confidence interval; HBV, hepatitis B virus; HCV, hepatitis C virus; WBC, white blood cells; Hb, hemoglobin; PLT, platelet count; PT, prothrombin time. *p = 0.025.

In-hospital mortality occurred in a total of 6 patients (5.8%). Sepsis was the most frequent non-bleeding-related cause of death (n = 3, 50%), followed by multiple organ failure (n = 2, 33.3%) (Table 7).

**Table 7:** Mortality and causes of death in the 2 groups of patients.

Characteristics	Group A (Child’s A patients) (n = 51)	Group B (Child’s B+C patients) (n = 51)	*p* Value
No. of deaths, n (%)	2 (3.9)	4 (7.8)	0.400
Cause of death			
Hypovolemic shock, n (%)	0	1 (2.0)	0.315
Sepsis, n (%)	1 (2.0)	2 (3.9)	0.558
Multiple organ failure, n (%)	1 (2.0)	1 (2.0)	1.000

## Discussion

One-third of all cirrhotic patients could experience variceal bleeding, with 70% recurrence and 20% mortality rates [17]. A previous study showed that 20% of cirrhotic patients developed bacterial infections upon hospital admission, and an additional 50% acquired infection during hospitalization [18]. The clinical benefit of using prophylactic antibiotics in reducing infections for cirrhotic patients who had variceal bleeding was statistically significant with either the fixed- or random-effects model, strengthening the evidence of the proposed effect [6]. All prophylactic regimens achieved this goal with strong potent antibiotics, which included either quinolone alone or quinolone given with amoxicillin-clavulanate, nonabsorbable antibiotics, and imipenem-cilastatin [2], [3], [7], [19]–[21]. The AASLD guidelines recommend oral norfloxacin (400 mg twice daily), intravenous ciprofloxacin, and intravenous ceftriaxone (1 g/day) as the preferred drugs [10], [22]. For patients with advanced cirrhosis (Child’s B and C), intravenous ceftriaxone is more effective than fluoroquinolone [9]. The reports on the effect of first-generation cephalosporins on cirrhotic patients with acute gastrointestinal hemorrhage are scarce. Very few studies clarify the effect of intravenous cefazolin prophylaxis on cirrhotic patients complicated with acute variceal bleeding. We believe that the result of the current pioneer study shows an important message about the potential benefit of prophylactic intravenous cefazolin–it may be not inferior to ceftriaxone in preventing infections and reducing rebleeding in Child’s A cirrhotic patients but not in those with advanced disease (Child’s B and C) after endoscopic interventions for acute variceal hemorrhage. Cirrhotic patients with more advanced disease who were prescribed with intravenous cefazolin developed infections and recurrent bleeding more frequently than those who were prescribed ceftriaxone.

Multiple confounding factors, such as Child’s B and C status, prothrombin time prolongation, hypoalbuminemia, and hyperbilirubinemia, were associated with infections; these could be the results of the advanced disease state. Age, hypoalbuminemia, and hyperbilirubinemia were identified as the independent predictors of bacterial infection, implying the poorer immune status of these patients. As for recurrent bleeding, there was no significant difference in the actuarial probability of remaining free of overall rebleeding between the Child’s A and Child’s B and C groups, despite a trend of more patients remaining free of overall rebleeding in those who were prescribed intravenous ceftriaxone, before subgroup analysis according to disease severity. This could be a source for misinterpretation because after we did subgroup analysis by disease severity (Child’s A and Child’s B and C patients), we observed more rebleeding cases in patients who received prophylactic cefazolin than in those who received ceftriaxone among Child’s B and C patients, but similar results were obtained for both antibiotics among Child’s A cirrhotic patients. In fact, the use of cefazolin instead of ceftriaxone was identified as an independent predictor of rebleeding in Child’s B and C cirrhotic patients on multivariate analysis. We believe that this is a potentially important message–that prophylactic intravenous cefazolin may not be inferior to ceftriaxone in preventing rebleeding among Child’s A cirrhotic patients. The use of cefazolin as a prophylactic antibiotic is seldom discussed concerning cirrhotic patients.

Increasing medical costs are having tremendous impact on the existing bad economy in most parts of the world. Therefore, a smart choice of effective antibiotics, preferably at a lower price, is important. The bottom line is that if this first-generation cephalosporin is proven effective for this particular disease group, its cost-effectiveness would be beneficial to medical care worldwide, especially for developing countries with poor medical resources. Additionally, the avoidance of antibiotic resistance must always be kept in mind. The use of cefazolin may be comparable to ceftriaxone in consideration of local quinolone resistance. Fernández et al. [5] showed that infections caused by gram-positive cocci were markedly increased by the extensive use of invasive procedures and long-term norfloxacin prophylaxis in the management of cirrhotic patients. Furthermore, the use of fluoroquinolone and extended-spectrum cephalosporins was reported to increase the incidence of extended-spectrum b-lactamase (ESBL)-producing bacteria, which was a major cause of nosocomial infections associated with high mortality [23]. Lee et al. [24] showed that the restriction of extended-spectrum cephalosporins significantly decreased the overall prevalence of ESBL production of *K. pneumoniae* and *E. coli* in children. The impact of a change in antibiotic policy was more evident in *K. pneumoniae* than in *E. coli*. Therefore, the use of cefazolin rather than third-generation cephalosporins may be a good choice in the economical point of view and may benefit public health care practices for the prevention of infection in Child’s A cirrhotic patients with acute variceal bleeding after endoscopic intervention.

The current study has some limitations. First, this is a single-center report; multicenter data may provide more convincing evidence on this issue. Second, this is a retrospective chart review study and the sample size is relatively small; therefore, bias may exist. A third limitation is the small sample size of Child’s C patients. These patients may have other problems causing poor survival, and therefore it is inevitable that Child’s C patients will be lost from the study. We therefore placed them in group B, as patients with advanced liver cirrhosis, for analysis. Then, we performed a case-control statistical analysis by entering the Child-Pugh scores into a regression model which provided more statistically convincing results. Child-Pugh-Turcotte scores were used as the primary metric throughout the study. The bottom line is that, although this study is hampered by the small sample size, this is the first study to identify that intravenous cefazolin may be sufficient as a prophylactic therapy for Child’s A cirrhotic patients. In fact, there was only 1 study, by Lin and colleagues [25], which showed that preprocedural and postprocedural administration of intravenous cefazolin, 1 g every 8 hours for 3 days, followed by oral cephalexin, 500 mg every 6 hours for 4 days, may prove safe and effective in reducing the infection rate in cirrhotic patients with upper gastrointestinal bleeding; however, its efficacy according to the different disease severity status was not analyzed in that study. Moreover, avoiding the use of strong antibiotics certainly helps in halting the already increasing antibiotic resistance problem worldwide. The much lower cost and easy availability of cefazolin may greatly reduce the burden on medical cost. However, this important message requires more large-scale prospective randomized studies for further validation.

In conclusion, this study suggests that prophylactic intravenous cefazolin may not be inferior to ceftriaxone in preventing infections and reducing rebleeding among Child’s A cirrhotic patients after endoscopic interventions for acute variceal bleeding, but prophylactic intravenous ceftriaxone yields better outcome among Child’s B and C patients.
